# Accuracy of portable spirometers in the diagnosis of chronic obstructive pulmonary disease A meta-analysis

**DOI:** 10.1038/s41533-022-00275-x

**Published:** 2022-04-19

**Authors:** Jiawei Zhou, Xiaomeng Li, Xingjian Wang, Na Yu, Wei Wang

**Affiliations:** Department of Respiratory and Critical Care Medicine, The First Hospital of China Medical University, 110000 Shenyang, China

**Keywords:** Physical examination, Chronic obstructive pulmonary disease, Health care economics

## Abstract

Portable spirometers has been approved for diagnosing chronic obstructive pulmonary disease (COPD). However, their diagnostic accuracy has not been reviewed. Therefore, the purpose of this study was to systematically evaluate the diagnostic value of portable spirometers in detecting COPD. A comprehensive literature search for relevant studies was conducted in PubMed, Embase, CNKI, Wan Fang, and Web of Science databases. Pooled sensitivity, specificity, summary receiver operating characteristic (SROC), area under the curve (AUC), and other related indices were calculated using the bivariate mixed-effect model. Subgroup analysis was performed to explore the source of heterogeneity. Thirty one studies were included in the meta-analysis. The pooled sensitivity, specificity, positive likelihood ratio (PLR), negative likelihood ratio (NLR), diagnostic ratio (DOR), SROC, and AUC of the SROC of portable spirometers were 0.85 (0.81–0.88), 0.85 (0.81–0.88), 5.6 (4.4–7.3), 0.18 (0.15–0.22), 31 (21–46) and 0.91 (0.89–0.94), respectively. Among the three commonly used types of portable spirometers, the accuracy of PIKO-6 was higher (0.95) than that of COPD-6 (0.91) and PEF (0.82). Subgroup analysis indicated that the accuracy of a multi-indices portable spirometer was higher than that of a single-index one (*P* < 0.05). In addition, portable spirometry performed by professional technicians in tertiary hospitals was more accurate than for those conducted by trained technicians in primary care facilities and communities (*P* < 0.05). Moreover, the accuracy of studies conducted in developing country was superior to developed country (*P* < 0.05). Portable spirometers have high accuracy in the diagnosis of COPD. Multi-index COPD-6 and PIKO-6 displayed higher accuracy than others. Standardized training of instrument operators should be considered to achieve reliable results.

## Introduction

Chronic obstructive pulmonary disease (COPD) is a respiratory condition characterized by persistent and progressive limitation in the airflow^1^. As one of the leading causes of disability and mortality globally, COPD accounted for nearly 3 million deaths in 2016^2^. Due to an increasing proportion of an aging population, coupled with the high cigarette smoking rate, the prevalence of COPD in China has increased by 67% in the last 10 years. The total number of COPD patients stands close to 100 million^3^. As a result, COPD has become one of the major public health challenges in China, with attendant heavy economic and social burden.

The 2021 Global Initiative for Chronic Obstructive Lung Disease (GOLD) document categorically points out that pulmonary function test (PFT) is the gold standard for COPD diagnosis. In addition, it states that PFT is the reference basis for grading the severity of COPD and guiding follow-up treatment; thus making it key in the diagnosis, treatment, and management of COPD^1^. Studies suggest that the prevalence of undiagnosed and underdiagnosed cases of COPD in primary care is substantial^4^, with most patients only getting diagnosed when they have already lost their lung function^5,6^. Gao and colleagues summarized the current status of the application of PFTs in China and decry that these tests are under-used in primary care. In fact, they noted, some primary care centers did not even provide these tests^7^. The low utilization of pulmonary function tests has been cited as the main reason for the failure to diagnose or underdiagnose COPD^8^. Conducting the traditional laboratory PFTs may not be feasible under primary care settings due prohibitive costs relating to acquisition, storage, and maintenance of the instruments besides the lack of professional technicians capable of operating the machines. However, if all patients suspected to have COPD are referred to tertiary hospitals for PFTs, this will increase their costs of seeking medical care.

Portable spirometers are attractive to use in clinical practice due to their affordability, portability, and easy-to-operate characteristics. Several studies have shown that the measurements obtained with the use of portable spirometers are highly consistent with those of traditional spirometers^9,10^. Thus, portable spirometers have gained prominence in medical practice and clinical research and can offer a suitable alternative for the early detection of COPD in resource-limited healthcare settings. The purpose of this systematic review and meta-analysis was to quantitatively evaluate the diagnostic accuracy and feasibility of the use of portable spirometers in the diagnosis of COPD.

## Methods

### Study identification and selection

Two authors searched independently from PubMed, Embase, CNKI, Wan Fang and the Web of Science databases. The search strategy was based on the following keywords and text words: (“COPD” OR “chronic obstructive pulmonary disease” AND (“portable spirometers” OR “handheld spirometry” OR “screening tool”) and related synonym extensions. The search time was from January 2000 to July 2021 with no language restrictions.

### Inclusion and exclusion criteria

For inclusion, Studies that designated the target disease as COPD. In addition, the individuals must have completed respiratory examinations using both a portable and traditional spirometer. Although peak flow meters are technically not spirometers, it was found to be used for COPD detection and pulmonary function evaluation in some studies. Therefore, we included peak flow meters in our study for comparing their sensitivity and specificity in COPD detection with other spirometers.

The following were excluded from the meta-analysis: (1) studies which did not report the numbers of true positives (TP), false positives (FP), true negatives (TN), and false negatives (FN) as well as any other relevant data for the construction of two-by-two contingency tables; (2) onference proceedings, expert forums, systematic reviews, translations, and such like articles.

### Data extraction

Data were extracted and cross-checked independently by two researchers. In case of any discrepancies, a third researcher was involved to adjudicate over the differences so that a common decision was reached. Information obtained from the studies include: (1) basic information such as author’s name(s), date of publication and sample size; (2) use of portable spirometers (including types, clinical setting, operators); (3) the number of TP, FP, FN, TN, and the threshold for identifying the positive values of the two tests. If more than one set of data (TP, FP, FN, and TN) was found, the set of data with the best diagnostic performance was chosen.

### Quality assessment

We divided the risk of bias of included studies into “high risk”, “low risk”, and “unclear risk”. The quality of each article included in this meta-analysis was assessed using the QUADAS-2 checklist as provided in Review Manager, version 5.2 (Copenhagen: The Nordic Cochrane Centre, The Cochrane Collaboration, 2012)^11^.

### Ethics statement

Procedures and experiment protocols were performed in accordance with the National Institute of Health Guide for Care and were approved by the Ethics Committee of China Medical University in accordance with the Declaration of Helsinki.

### Statistical analysis

Forest plots of sensitivity and specificity were constructed using Review Manager, version 5.2. These plots were used to visually explore the diagnostic accuracy of each test. Statistical analyses was conducted using Stata, version 13.1 (Stata-Corp, College Station, Texas, USA). The “midas” command was used to fit the bivariate mixed-effects model to estimate coefficients and the variable-covariate matrix. The same was used to calculate the pooled sensitivity, specificity, positive likelihood ratio (PLR), negative likelihood ratio (NLR), and diagnostic odds ratio (DOR) with 95% confidence intervals (CI) for each of the included studies. A summary receiver operating characteristic curve (SROC) was drawn, and the area under the curve (AUC) was calculated to describe and compare the accuracy of portable spirometers in the diagnosis of COPD. The accuracy of the diagnostic test was evaluated according to the value of the AUC, which was divided into five parts: non-informative (AUC = 0.5), less accurate (0.5 < AUC < 0.7), moderately accurate (0.7 < AUC < 0.9), highly accurate (0.9 < AUC < 1), and perfect tests (AUC = 1)^12^.

The I^2^ test was used to estimate the heterogeneity of the included studies contributing to the pooled estimate. After the influence of the threshold effect was excluded by Meta-Disc version 1.4 (Unit of Clinical Biostatistics Team of the Ramón y Cajal Hospital, Madrid, Spain). Random effect model was used to provide a conservative estimate of statistics, afterwards, potential heterogeneity was explored using subgroup analysis which both were conducted using STATA 13.1 Software. The subgroup analyses included the grouping based on threshold selection method (fixed value or the cutoff value), the type of portable spirometer indicators (multi-index or single index), country (developed countries or developing countries), study setting (hospital or normal population), type of executive place (tertiary hospitals or primary cares and communities), population (non COPD or the whole crowd), and the year of publication (2000–2016 or 2017–2021). The level of significance ɑ was adjusted for multiple comparisons. Sensitivity analyses were performed to determine the reliability of the results, and Deeks’ funnel plot was used to detect publication bias. The results were considered statistically significant when *P* < 0.05.

## Results

### Search results

**Fig. 1:**
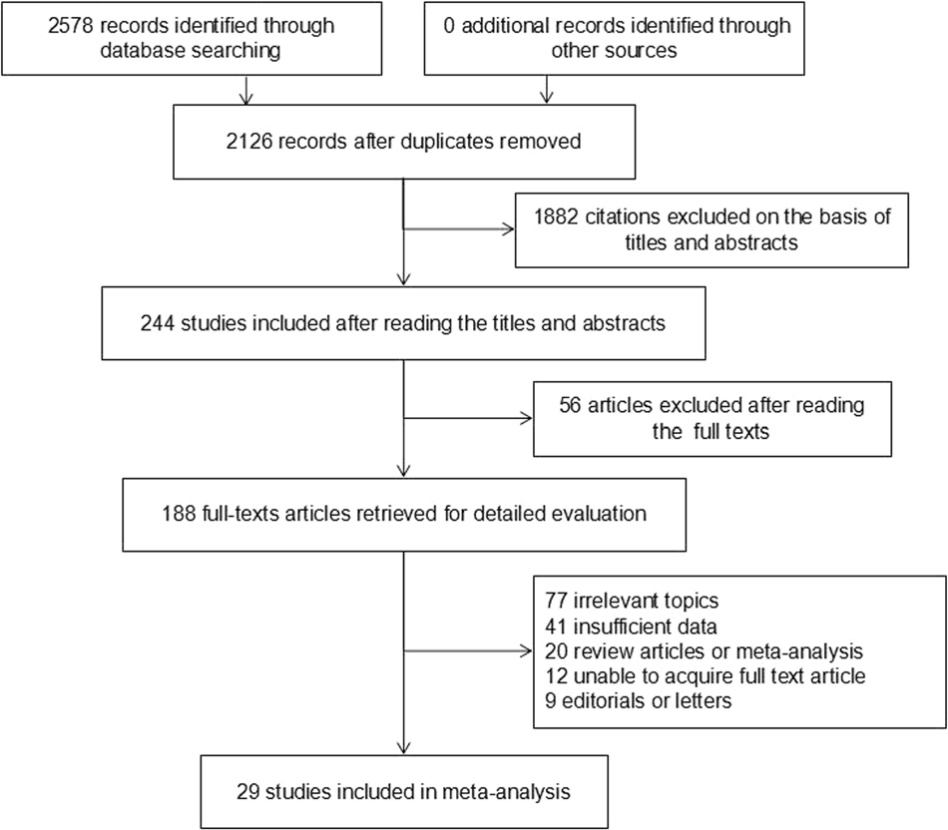
Studies selection for meta-analysis. COPD chronic obstructive pulmonary disease.

A total of 2578 related articles were obtained in the initial database inspection according to the previously described search strategy (Fig. 1). After removal of duplicate publications, title, and abstract screening, 244 articles were identified as being potentially suitable for inclusion. Subsequently, 188 articles were selected after reading the full text. Finally, 29 articles were included in the meta-analysis after applying the inclusion and exclusion criteria (including 2 in Chinese and 27 in English). One of the articles used two portable spirometers in the same population, and one of the articles conducted one portable spirometer in two different population, for these reason, it was split into two independent studies in the subsequent analysis. Consequently, 31 studies were included in this meta-analysis.

The studies included in this meta-analysis were conducted in 15 countries (6 in China, 5 in Spain, 3 in the UK, 3 in Japan, 2 in Australia, 2 in Korea, 2 in India, and 1 in the United Arab Emirates, Germany, Netherlands, Croatia, Sweden, Iran, Malaysia, and Greece, respectively), in tertiary hospitals (10 articles), primary care units or community settings (21 pieces), and utilized nine types of portable spirometers. These devices were COPD-6 (*n* = 14), PIKO-6 (*n* = 6), PEF (*n* = 4), Hi-Checker (*n* = 2), and IQ-Spiro (*n* = 1), Medikro SpiroStar (*n* = 1), MS01 Micro spirometer (*n* = 1), SP10BT (*n* = 1), Spirobank Smart (*n* = 1) (Table 1).

**Table 1:** Characteristics of studies included in the meta-analysis.

Author and year	Country	Setting	Study designs	Portable spirometer	Test threshold of portable spirometers	Definition of airflow obstruction	Portable spirometers operator	Inclusion and exclusion criteria	Sample size (male)	Age (years, mean ±SD)
Chen G 2018^10^	China	1 tertiary hospital	randomized	IQ-spiro	Cut-off value unclear-FEV_1_/FEV_6_<0.74	post-FEV_1_/FVC<0.70	Professional technician	Subjects who visited a tertiary hospital	159 (-)	55±14.7
Chen S 2021^37^	China	8 primary cares	cross-sectional study	COPD-6	Cut-off value unclear-FEV_1_/FVC<0.77	post-FEV_1_/FVC<0.70	Trained physician	Aged ≧ 40 years	1487 (-)	-
Dickens AP 2020^38^	UK	71 general practices	case-control	COPD-6	Cut-off value Pre-FEV_1_/FEV_6_<0.78	post-FEV_1_/FVC<LLN	Trained research assistants	aged≧40, who either had previously clinically diagnosed COPD or had reported chronic respiratory symptoms.	544 (349)	69.6±9.1
Figueira Goncalves JM 2017^39^	Spain	1 tertiary hospital	cross-sectional observational study	COPD-6	Cut-off value Pre-FEV_1_/FEV_6_<0.75	pre-FEV_1_/FVC<0.70	Professional technician	patients referred to laboratory for respiratory functional tests	233 (133)	59±15
Frith P 2011^40^	Australia	4 primary care practices	prospective, multicenter	PiKo-6	Cut-off value preFEV_1_/FEV_6_<0.75	post-FEV_1_/FVC<0.70	Trained nurse or general practitioner (GP)	current or former smokers, aged > 50 years, no previous diagnosis of obstructive lung disease, and no treatment for obstructive lung disease in the past year.	204 (69)	61±8
Frith P 2011*^40^	Australia	4 primary care practices	prospective, multicenter	PiKo-6	Cut-off value Pre-FEV_1_/FEV_6_<0.75	post-FEV_1_/FVC<0.70	Trained nurse or general practitioner (GP)	current and former smokers aged >50 years, a previous diagnosis of or treatment for asthma, and no previous diagnosis of COPD.	93 (54)	62±8.8
Hidalgo Sierra V 2018^41^	Spain	2 primary care centers*	-	PiKo-6	Cut-off value unclear-FEV_1_/FEV_6_≦0.70	post-FEV_1_/FVC<0.70	Professional technician*	aged ≧40 years, a pack-year index (PYI)≧10, and typical symptoms, such as cough, expectoration and dyspnea, and with no previous diagnosis of COPD	155 (111)	63±14
Hwang YI 2021^42^	Korea	5 tertiary hospitals	-	COPD-6	Cut-off value pre-FEV_1_/FEV_6_<0.73	post-FEV_1_/FVC<0.70	Professional technician	Aged ≧ 40 years; respiratory symptoms and PYI≧10 pack-years. Subjects who had a history of disease such as tuberculous sequalae, bronchiectasis, asthma, and lung cancer that might influence pulmonary function tests were excluded.	290 (-)	63.1 ± 11.0
Kim JK 2016^43^	Korea	9 primary clinics	prospective cohort study	COPD-6	Cut-off value preFEV_1_/FEV_6_≦0.77	post-FEV_1_/FVC<0.70	primary care physicians	Subjects who visited a primary clinic complaining of respiratory symptoms and aged ≧40 years, PYI≧10 irrespective of their current smoking state and had no previous diagnosis of COPD. Patients with a history of disease that might have influenced spirometry results, such as tuberculosis-destroyed lungs, bronchiectasis, asthma, or lung cancer were excluded.	190 (-)	60.3±10.6
Kobayashi S 2017^44^	Japan	16 primary care clinics and 4 hospitals	prospective multi-center, observational study	Hi-Checker	Fixed value unclear-FEV_1_/FEV_6_≦0.75	post-FEV_1_/FVC<0.70	primary care physicians	Patients > 40 years of age who received outpatient care for chronic disease at primary care clinics Patients with known chronic respiratory diseases, including asthma and COPD, and patients suffering from acute respiratory symptoms were excluded.	110 (91)	68.5±0.8
Labor M 2016^9^	Croatia	26 general practitioners’ office	prospective cohort study	COPD-6	Cut-off value unclear-FEV_1_/FEV_6_≦0.78	post-FEV_1_/FVC<0.70	Trained GPs	written consent. aged 40–65 years with a smoking history of PYI≧2; with no previous diagnosis of COPD.	227 (112)	52.5±6.8
Li XF 2020^45^	China	1 district hospitals and ten primary care centers.	-	SP10BT	Cut-off value post-FEV_1_/FVC<0.7 and FVC>80%pred	post-FEV_1_/FVC<0.70 and FVC<80%pred	2 trained primary care physicians	aged > 40 years, typical symptoms such as chronic cough expectoration, or asthma, and risk factor exposure history	252 (182)	65.7±10.1
Lin CH 2021^46^	Taiwan, China	26 outpatient clinics	prospective multi-center,	Spirobank Smart	Cut-off value pre-FEV_1_/FVC<0.74	post-FEV_1_/FVC<0.70	trained nurses and physicians	Aged ≧ 40 years, PYI≧10 pack-years., with chronic respiratory disease and no previous diagnosis of COPD.	370 (349)	60.9±9.7
Llordes M 2017^47^	Spain	8 primary care centers	-	COPD-6	Cut-off value unclear-FEV_1_/FEV_6_≦0.78	post-FEV_1_/FVC<0.70	Trained primary care physician, a nurse or a technician	Aged ≧ 40 years with a smoking history of PYI≧1; with no previous diagnosis of COPD and attended the primary care centers for any reason	407 (265)	57.4±8.9
Mahboub B 2014^22^	United Arab Emirates	5 primary cares, 2 large shopping malls and 1 industrial city	cross sectional study	PEF	Fixed value Pre-PEF<2.2L(s*m^2^)	pre-FEV_1_/FVC<0.70	Trained primary care physicians or nurses	Aged ≧ 40 years	525 (358)	49.6±4.1
Ng, S. C. 2017^48^	Malaysia	Medical Outpatient Department and health care clinics	cross-sectional study	COPD-6	Cut-off value post-FEV_1_/FEV_6_<0.75	post-FEV_1_/FVC<0.70	Trained stuff	Aged ≧50 years; history of dyspnoea; history of chronic cough or chronic sputum production; history of exposure to risk factors; and any smoker even in the absence of above symptoms.	117(101)	67.38±11.58
Nishimura K 2011^49^	Japan	1 tertiary hospital	-	Hi-Checker	Cut-off value unclear-FEV_1_/FEV_6_<0.746	post-FEV_1_/FVC<0.70	Professional technician	industrial workers who underwent annual health checks	312 (312)	55±9.4
Represas CR 2010^50^	Spain	1 tertiary hospital	prospective, descriptive transversal study	COPD-6	Cut-off value unclear-FEV_1_/FEV_6_<0.77	unclear-FEV_1_/FVC<0.70	Professional technician	those who attended pulmonary function laboratory for functional respiratory tests	162 (95)	56±16
Represas-Represas C 2016^51^	Spain	8 primary care centers, 15 community pharmacies and 4 emergency services	prospective, multi-cohort study	COPD-6	Cut-off value preFEV_1_/FEV_6_<0.80	post-FEV_1_/FVC<0.70	Trained primary care physicians or nurses	Aged ≧40 years, with a smoking history of PYI≧10, and symptoms suggestive of COPD. Individuals who had already been diagnosed with a respiratory disease were excluded.	362 (224)	55.4±9.9
Ronaldson SJ 2018^23^	UK	general practices	prospective case-finding stud	PEF	Fixed value Pre-PEF<80%pred	post-FEV_1_/FVC<0.70 and FEV_1_%<80%pred or FEV_1_%>80%pred with at least 1 symptom	Trained nurses in primary care	aged≧35; current smokers, including those who had comorbidities, such as COPD or asthma	216 (109)	53.4±11.0
Ronaldson SJ 2018*^23^	UK	general practice	prospective case-finding stud	MS01 Micro spirometer	Fixed value FEV_1_/FVC<0.7, FEV_1_<80%pred, or FVC<80%pred	post-FEV_1_/FVC<0.70 and FEV_1_%<80%pred or FEV_1_%>80%pred with at least 1 symptom	Trained nurses in primary care	aged≧35; current smokers, including those who had comorbidities, such as COPD or asthma	216 (109)	53.4±11.0
Sami R 2020^52^	Iran	1 tertiary hospital	cross-sectional descriptive study	COPD-6	Cut-off value post-FEV_1_/FVC<0.72	post-FEV_1_/FVC<0.70	Professional technician	Aged ≧ 40 years; PYI≧10 pack-years; symptoms suggestive of COPD. Patients with previously diagnosed respiratory diseases were excluded.	122 (-)	53.2±9.0
Schneider A 2009^53^	Germany	10 primary care centers	cross-sectional study	Medikro SpiroStar	Fixed value post-FEV_1_/FVC<0.70and FVC>80%pred	post-FEV_1_/FVC≦0.70 and/or FEV_1_<80%pred	Trained primary care physicians and practice assistants	Complaints suggestive of obstructive airway disease (OAD), had not been diagnosed previously for OAD and had not received any previous anti-obstructive medicine.	219 (92)	43.8±15.6
Sichletidis L 2011^54^	Greece	Two Primary cares Centers	-	PiKo-6	Fixed value post-FEV_1_/FEV_6_<0.70 and/or FEV1<80%	post-FEV_1_/FVC<0.70	Trained primary care general practitioners	Aged >40years. Medically confirmed diagnosis of an obstructive lung disease (e.g. COPD, asthma, bronchiectasis) and any other pulmonary disease (e.g. tuberculosis, interstitial lung disease, lung cancer), thoracic surgery in the previous 6 months or acute respiratory infection were excluded.	1078 (616)	65.3±11.4
Thorat YT 2017^24^	India	Specialized Hospital	cross-sectional study	PEF	Cut-off value pre-PEF<80%pred	post-FEV_1_/FVC<0.70	Professional technician	Adult patients with respiratory complains. Patients with history of pulmonary tuberculosis, and those with contra-indications for spirometry, and also pregnant and nursing mothers were excluded.	189 (111)	51±17
Thorat YT 2017^*24^	India	Specialized Hospital	cross-sectional study	COPD-6	Cut-off value preFEV_1_/FEV_6_<0.75	post-FEV_1_/FVC<0.70	Professional technician	Adult patients with respiratory complains. Patients with history of pulmonary tuberculosis, and those with contra-indications for spirometry, and also pregnant and nursing mothers were excluded.	189 (111)	51±17
Thorn J 2012^55^	Sweden	Twenty-one urban and rural Primary cares Centers	-	COPD-6	Cut-off value preFEV_1_/FEV_6_<0.73	post-FEV_1_/FVC<0.70	Trained nurse	Aged 45–85 years; with a smoking history of at least 15 pack-years	305 (132)	61.2±8.4
Tian J 2012^25^	China	Community settings	cluster Sampling; Prospective study	PEF	Fixed value pre-PEF<80%pred	post-FEV_1_/FVC<0.70	Trained primary care physicians or nurses	Aged ≧40years	3379 (1400)	-
Toda R 2009^56^	Japan	1 tertiary hospital	-	PiKo-6	Cut-off value pre-FEV_1_/FEV_6_<0.749	pre-FEV_1_/FVC<0.70	Professional technician	subjects including non-smokers, smokers, COPD, bronchial asthma, and interstitial lung diseases.	768 (402)	57.8±0.6
van den Bemt L2014^57^	Nether lands.	several sites for general practitioners	randomized cross-sectional diagnostic study	PiKo-6	Fixed value pre-FEV_1_/FEV_6_<0.73	post-FEV_1_/FVC<0.70	Trained primary care physicians	Aged ≧50 years; current or former smokers (⩾1 pack year) with no previous diagnosis of COPD	104 (62)	-
Wang XY 2018^58^	China	1 community	-	COPD-6	Fixed value pre-FEV_1_/FEV_6_<0.70	post-FEV_1_/FVC<0.70	Trained technician	Aged 40-75 years	475 (134)	57.78±9.18

^*^In this study, both portable spirometers and traditional laboratory PFT were conducted by the high-resource medical institutions.

COPD, chronic obstructive pulmonary disease; FEV_1_,forced expiratory volume in 1 s; FEV_6_, forced expiratory volume in 6 s; FVC, forced vital capacity; GP, general practitioner; PYI, pack year index; SD, Standard Deviation.

### Literature bias risk assessment and publication bias

**Fig. 2:**
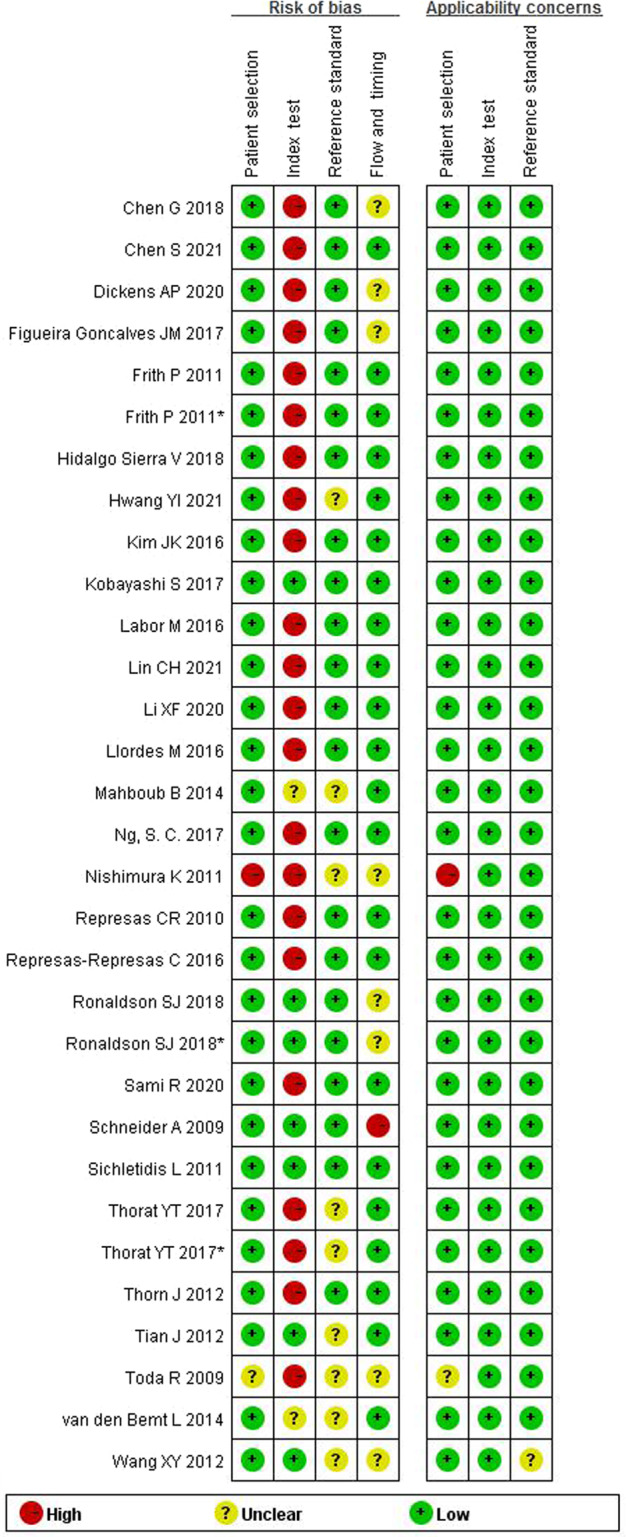
Summary map of risk of bias across domains of the included studied. Using QUADAS-2 tool. Key domains: patient selection; index test; reference standard; study flow and timing. The risk of bias is indicated by three colors, red for high risk of bias, yellow for unclear risk of bias, and green for low risk of bias.

**Fig. 3:**
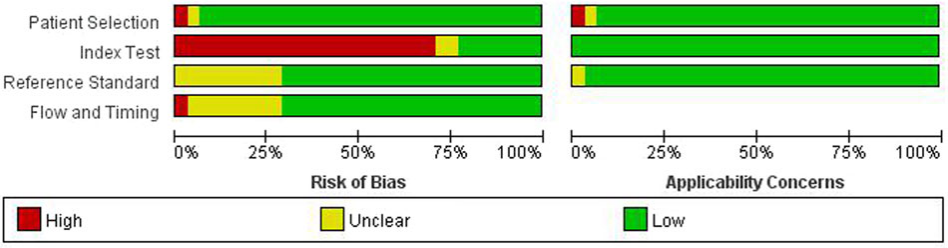
Risk of bias and corresponding applicability concerns across included studies. Using QUADAS-2 tool. Key domains: patient selection; index test; reference standard; flow and timing. The risk of bias is indicated by three colors, red for high risk of bias, yellow for unclear risk of bias, and green for low risk of bias.

Generally, the quality of the included studies ranged between medium to high. Assessment using QUADAS-2 tools found that “patient selection” and “flow and timing” parts of tools were clear for most studies. The risk of bias mainly arose from the selection method of the threshold of the index test and the lack of a strict blinding method between the index test and the reference test (Figs. 2 and 3).

**Fig. 4:**
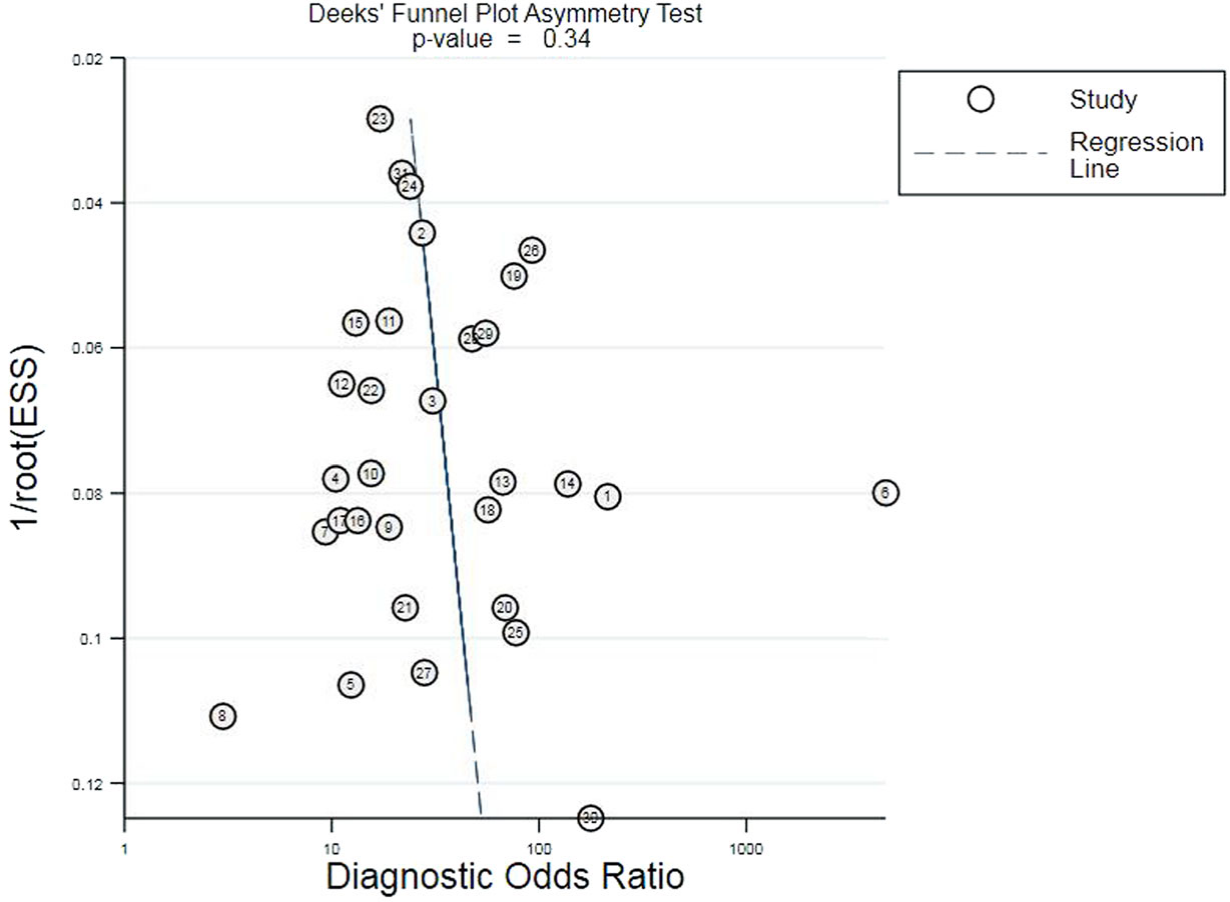
Deeks’ funnel plot asymmetry test for evaluation of publication bias. The closer the angle between the regression line of the Deeks’ funnel plot and the horizontal axis (x) is to 90, the less likely it is to suggest that there is publication bias.

There was no significant publication bias as determined by the Deeks’ funnel chart, which showed that the angle between the regression line and the horizontal axis was close to 90° (*P* = 0.34) (Fig. 4).

### Diagnostic accuracy of spirometry

**Fig. 5:**
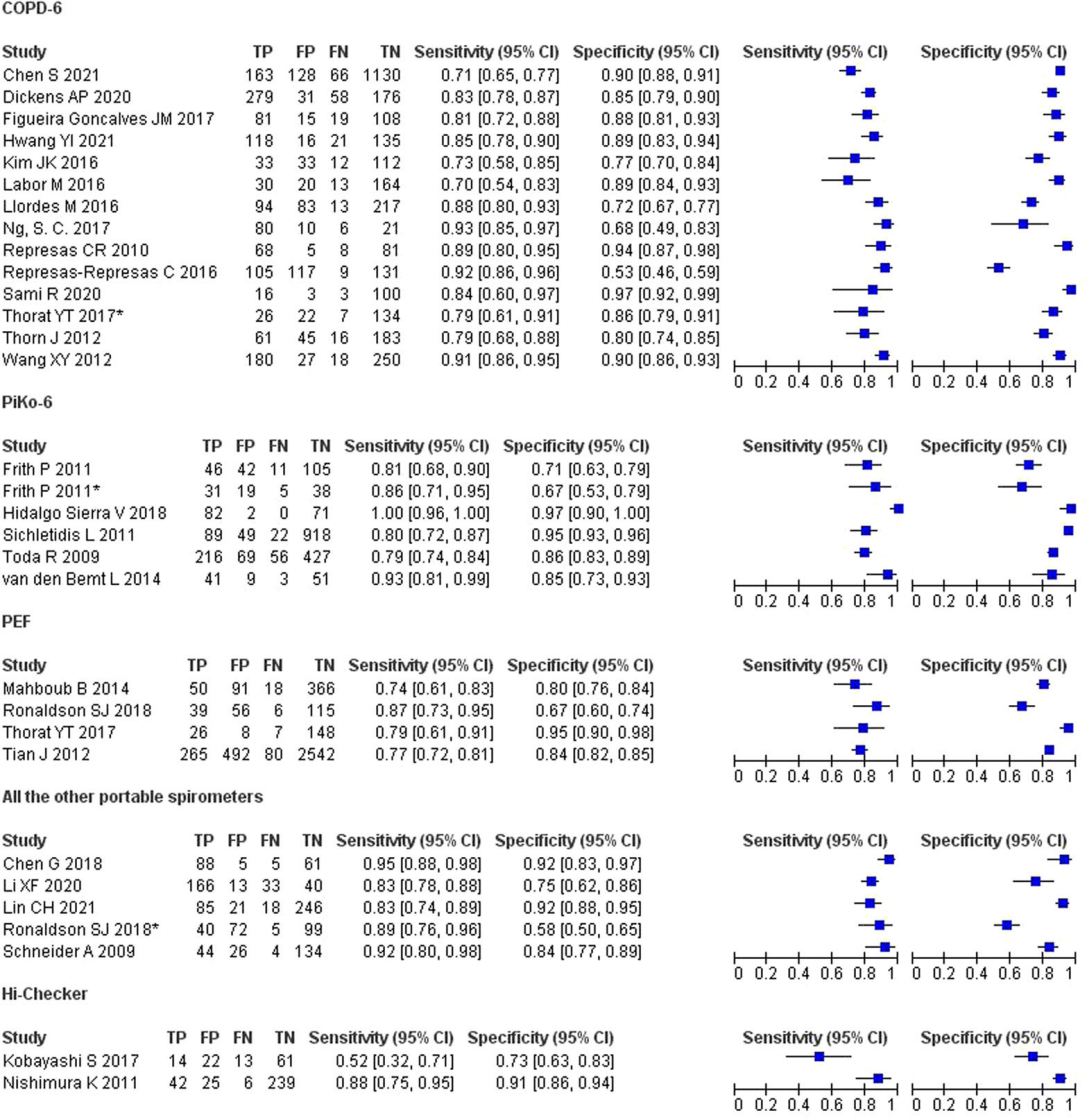
Forest plot of sensitivity and specificity of each screening test. TP true positive, FP false positive, FN false negative, TN true negative.

**Fig. 6:**
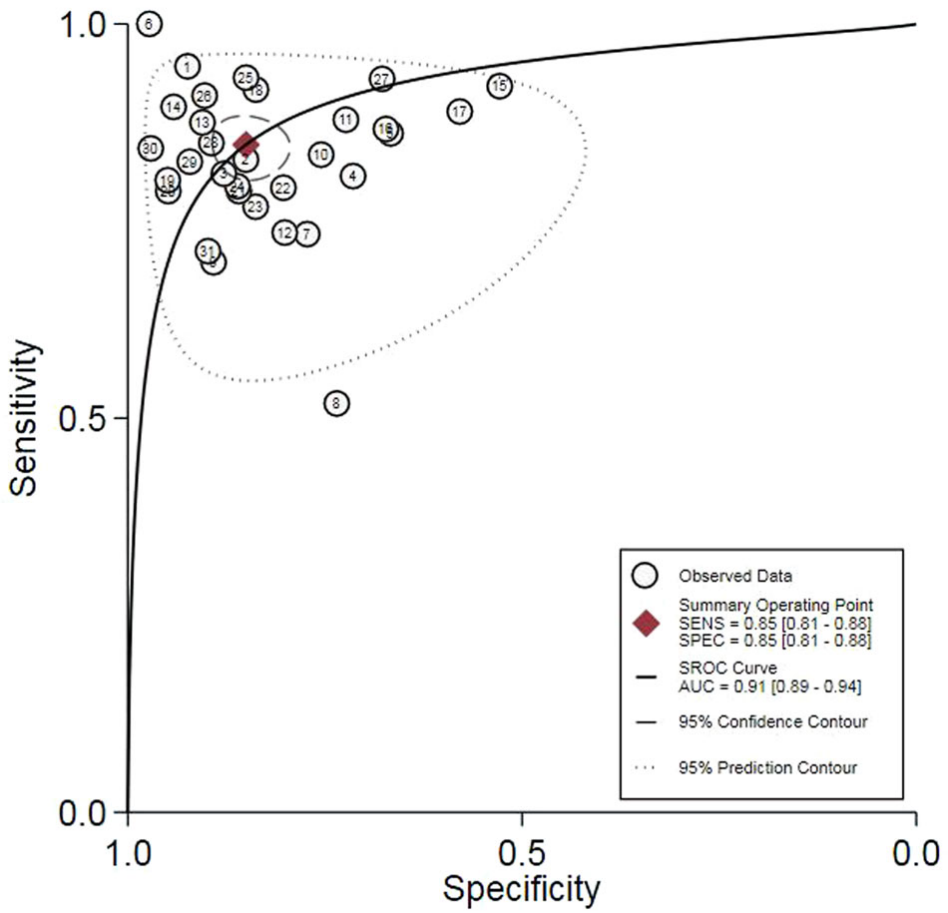
Bivariate summary estimates of sensitivity and specificity, with corresponding 95% confidence ellipse around the mean values, for all studies. SENS sensitivity, SPEC specificity, SROC summary receiver operating characteristic, AUC area under the curve.

The results of TP, FP, FN, and TN in the diagnosis of COPD in each study are shown in (Fig. 5). The Spearman correlation coefficient between the logit of sensitivity and logit of 1-specificity was 0.011 (*P* = 0.955), indicating that there was no threshold effect in the study. However, the I^2^ values were high (I^2^ = 99%, *P* < 0.01). We chose the random-effects model to conservatively estimate the summary statistics. The results show that the pooled sensitivity, specificity, PLR, NLR, and DOR with 95% CI are 0.85 (0.81–0.88), 0.85 (0.81–0.88), 5.6 (4.4–7.3), 0.18 (0.15–0.22), and 31 (21–46), respectively. The area under the SROC (AUC) was 0.91 (0.89–0.94) showing that the accuracy of the portable spirometer is 91% and is very close to the reference test (Fig. 6).

### Subgroup analysis

**Fig. 7:**
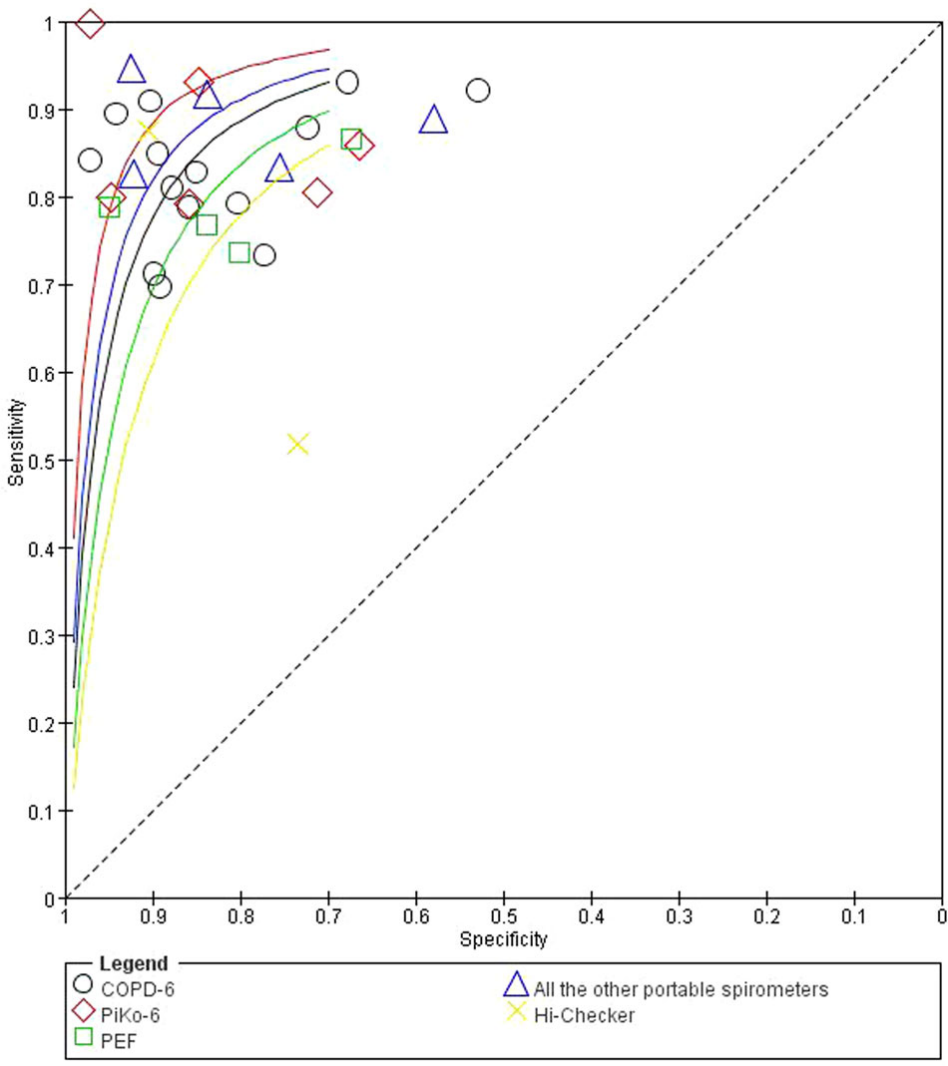
The SROC of portable spirometers classified by type. All the other portable spirometers including IQ-spiro, SP10BT, Medikro SpiroStar, MS01 Micro spirometer and Spirobank Smart.

The outcomes of the subgroup analyses are summarized in Table 2. PIKO-6 had the highest diagnostic accuracy with the area under the SROC (AUC) of 0.95 (0.92–0.96). AUC value for COPD-6 was 0.91 (0.88–0.93), and for PEF it was 0.82 (0.78–0.85). There were statistically significant differences in the diagnostic accuracy indices among COPD-6, PIKO-6, and PEF (P all < 0.0167) after adjusting the level of significance ɑ due to multiple comparisons (Fig. 7). According to the classification of detection indicators, portable spirometers with FEV_1_/FEV_6_ showed the area under the SROC (AUC) was 0.92 (0.90–0.94). There were statistically significant differences in the diagnostic accuracy indices between PEF and FEV_1_/FEV_6_ (*P* < 0.001), and between FEV_1_/FEV_6_ and FEV_1_/FVC (*P* = 0.007) after adjusting the level of significance ɑ due to multiple comparisons.

**Table 2:** Subgroup analysis of all included studies.

Factor	Studies	Sensitivity (95% CI)	I^2^	Model used	Specificity (95% CI)	I^2^	Model used	SROC (95% CI)
All studies	31	0.85 (0.81–0.88)	77.87%	Random	0.85 (0.81–0.88)	95.48%	Random	0.91 (0.89–0.94)
Type of the device								
COPD-6	14	0.84 (0.80–0.88)	79.22%	Random	0.85 (0.79–0.90)	95.85%	Random	0.91 (0.88–0.93)
Piko-6	6	0.89 (0.76–0.96)	84.17%	Random	0.88 (0.75–0.94)	96.11%	Random	0.95 (0.92–0.96)
PEF	4	0.77 (0.73–0.81)	1.13%	Fixed	0.83 (0.71–0.91)	94.04%	Random	0.82 (0.78–0.85)
Hi-checker	2	0.75 (0.63–0.84)	91.20%	Random	0.86 (0.82–0.90)	92.80%	Random	–
IQ-spiro	1	0.94	–	–	0.92	–	–	–
SP10BT	1	0.83	–	–	0.75	–	–	–
Medikro SpiroStar	1	0.92	–	–	0.84	–	–	–
MS01 Micro spirometer	1	0.89	–	–	0.58	–	–	–
Spirobank Smart	1	0.83	–	–	0.92	–	–	–
Detection indicators								
PEF	4	0.77 (0.73–0.81)	1.13%	Fixed	0.83 (0.71–0.91)	94.04%	Random	0.82 (0.78–0.85)
FEV_1_/FVC	4	0.85 (0.81–0.88)	6.47%	Fixed	0.80 (0.65–0.90)	96.14%	Random	0.87 (0.84–0.90)
FEV_1_/FEV_6_	23	0.85 (0.81–0.89)	82.09%	Random	0.86 (0.81–0.90)	95.54%	Random	0.92 (0.90–0.94)
Threshold selection method								
Fixed	4	0.83 (0.65–0.92)	90.39%	Random	0.89 (0.79–0.94)	94.40%	Random	0.93 (0.90–0.95)
Cutoff	19	0.86 (0.81–0.89)	80.62%	Random	0.85 (0.80–0.90)	95.43%	Random	0.92 (0.89–0.94)
*P*		0.673			0.385			0.581
Type of indicators								
Multi-index	27	0.85 (0.82–0.88)	79.21%	Random	0.85 (0.81–0.89)	95.60%	Random	0.92 (0.89–0.94)
Single index	4	0.77 (0.73–0.81)	1.13%	Fixed	0.83 (0.71–0.91)	94.04%	Random	0.82 (0.78–0.85)
*P*		<0.001			0.716			<0.001
Country								
Developed	23	0.85 (0.81–0.88)	73.60%	Random	0.83 (0.78–0.87)	95.52%	Random	0.90 (0.88–0.93)
Developing	8	0.84 (0.77–0.89)	88.47%	Random	0.90 (0.85–0.93)	96.57%	Random	0.94 (0.91–0.95)
*P*		0.778			0.023			0.015
Executive place								
Tertiary hospital	10	0.89 (0.83–0.93)	81.27%	Random	0.92 (0.89–0.95)	82.86%	Random	0.96 (0.94–0.97)
Primary care/community	21	0.83 (0.79–0.87)	76.98%	Random	0.80 (0.75–0.85)	95.97%	Random	0.89 (0.86–0.91)
*P*		0.066			<0.001			<0.001
Study setting								
Hospital-based	27	0.85 (0.81–0.88)	77.64%	Random	0.85 (0.80–0.89)	95.81%	Random	0.91 (0.89–0.93)
Population-based	4	0.85 (0.78–0.90)	86.53%	Random	0.87 (0.84–0.90)	94.79%	Random	0.93 (0.90–0.95)
*P*		0.999			0.469			0.222
Population								
No COPD	13	0.86 (0.78–0.91)	80.71%	Random	0.87 (0.81–0.92)	94.67%	Random	0.93 (0.90–0.95)
The whole crowd	18	0.84 (0.81–0.87)	76.22%	Random	0.83 (0.78–0.87)	95.81%	Random	0.90 (0.87–0.93)
*P*		0.584			0.271			0.133
Publication year								
2000–2016	15	0.83 (0.79–0.86)	66.10%	Random	0.83 (0.77–0.87)	95.94%	Random	0.89 (0.86–0.92)
2017–2021	16	0.86 (0.80–0.90)	83.90%	Random	0.87 (0.81–0.91)	95.02%	Random	0.92 (0.89–0.95)
*P*		0.336			0.267			0.178

The study divides countries into developed and developing countries according to HDI. When HDI > 0.80, it is classified as a developed country and if otherwise, developing country^59^.

*SROC* summary receiver operating characteristic curve.

^a^Owing to the complexity of the bivariate model and the limited number of studies, the groups with *n* ≥ 4 were pooled using a bivariate model. The remaining data were pooled by univariate random-effects logistic regression model. We also tested for the difference in the sensitivity or specificity between the two groups using the bivariate model. The AUC difference in the area under the SROC between the groups was obtained by Z test.

Based on the subgroup analyses, sources of heterogeneity could not be traced with regard to the threshold selection method, study setting, population, and year of publication. However, the source of heterogeneity can be attributed to the place of execution, the type of portable spirometer classified by indicators and the country sorted by Human Development Index (HDI). When classified by indicator type, the area under the SROC (AUC) was 0.92 (0.89–0.94) for the multi-indices group and 0.82 (0.78–0.85) for the single-index group. This difference was statistically significant both in sensitivity and AUC (*P* all < 0.001). When we grouped studies by the clinical setting in which the spirometry was conducted, the area under the SROC (AUC) of the tertiary hospital group was 0.96 (0.94–0.97), and 0.89 (0.86–0.91) for the primary care and community group. Statistically significant differences in AUC and specificity were noted between these groups (*P* all < 0.001). When we grouped studies by country, the area under the SROC (AUC) of the developed country was 0.90 (0.88–0.93), and 0.94 (0.91–0.95) for the developing country statistically significant differences in AUC (*P* = 0.015) and specificity (*P* = 0.023) were noted between these groups.

## Discussion

COPD has been widely underdiagnosed so far. PFT has been recommended as the gold standard for COPD diagnosis and monitoring^1^. However, such tests are not readily available or applied to all patients in need, leading to the absence of standard diagnosis and treatment, and subsequently the deterioration of COPD^13^. A decision-analytic model conducted by Qu S et al. showed that portable spirometer is likely the optimal option in the early screening and follow-up of patients in China^14^. In addition, multiple screening questionnaires have been developed as active case-finding tools to identify potential COPD patients in primary care^15^. Haroon^16^ compared the diagnostic accuracy of screening tests in primary care in 2015, finding that portable spirometers had a sensitivity of 79.9% (74.2–84.7%) and a specificity of 84.4% (68.9–93.0%). He concluded that portable spirometers demonstrated higher test accuracy than questionnaires for COPD screening in primary care. However, There were only three relevant references in Haroon’s study concerning portable spirometers, which was too small to further analyze the clinical application effects and influencing factors of portable spirometers. To address this gap, we performed a more detailed and comprehensive meta-analysis in this field by including 31 studies for systematic evaluation and quantitative analysis. Across the studies, nine types of portable spirometers were used under three different kinds of medical environments. We excluded the influence of threshold effects and used random-effects models to pool the data. The results show that the area under the SROC (AUC) of 0.91 indicate that the portable spirometer has high accuracy and can be used as an alternative for traditional pulmonary function tests in COPD screening, primary diagnosis and subsequent monitoring. COPD-6, PIKO-6, and PEF are three commonly used portable spirometers in clinical practice. From a diagnostic accuracy perspective, PIKO-6 has the highest diagnostic accuracy rate (95%), followed by COPD-6 (91%) and PEF (82%) with statistically significant difference among them (*P* < 0.05).

The heterogeneity in these studies was explored by subgroup analyses. According to the GOLD guideline, post-bronchodilator FEV_1_/FVC < 0.70 is the criterion for diagnosing COPD^1^. However, a qualified FVC measurement based on the ATS guideline has high requirements for the subject and the operator. A growing number of studies indicated that the FEV_1_/FEV_6_ could be served as an alternative choice for FEV_1_/FVC^17–19^. FEV_6_ is more accessible to measure than FVC and reduces the probability of spirometry complications. Several portable spirometers, such as COPD-6, PIKO-6, were designed to measure FEV_6_ instead of the original FVC. As mentioned above, some studies in this meta-analysis defined the fixed FEV_1_/FEV_6_ < 0.70 as airflow obstruction. However, considering that the reference formula for FEV6 was originated from the lung function database of the Third National Health and Nutrition Examination Survey(NHANES III) conducted in American^20^. There is still a debate on whether its application to the population of other countries and regions will make a difference. Some studies have been modified its ratio from the fixed value to an optimal cutoff value based on the national population. Therefore, we compared the diagnostic accuracy of portable spirometer using the fixed value with the cutoff value. Our study found that differences in the diagnostic accuracy of portable spirometers have nothing to do with the threshold selection method. Although the cutoff value can get the best diagnostic effect, a fixed value can also be acceptable if applied to primary diagnosis or community screening. Besides, we also investigated the diagnostic accuracy of all spirometers using FEV_1_/FEV_6_ ratio. Our pooled estimates showed a diagnostic accuracy of 92% with FEV_1_/FEV_6_, compared with the gold standard using FEV_1_/FVC. This study showed that the FEV_1_/FEV_6_ could be served as an alternative choice for FEV_1_/FVC for the diagnosis of COPD. Still, Soares et al. compared the sensitivity of FEV_1_/FEV_6_ with that of FEV_1_/FVC and concluded that although FEV_1_/FEV_6_ showed a good sensitivity of 85.6–95%, when it comes to mild airway obstruction, the sensitivity will be decreased^21^.

We found that the heterogeneity in test accuracy between studies was likely to arise from differences in the type of portable spirometer index, executive place, and country, but not in the threshold selection method, study setting, population, or year of publication.

Portable spirometers, for example, PIKO-6 and COPD-6 can give multiple respiratory function indicators, such as FEV_1_, FEV_6_, and FVC. These indicators can help in the diagnosis of patients with COPD, as well as in estimating the severity of the disease, and then guiding the choice of an appropriate treatment plan. However, there are still some studies^22–25^ that applied PEF for screening and diagnosis of COPD. As single-index spirometry, PEF has been widely used to diagnose and monitor asthma patients^26,27^. Liu YN^28^ and Jackson et al.^29^ concluded that PEF has extremely high sensitivity (98.5–100%) in screening moderate to severe COPD patients. To assess whether the type of indicators impacts the diagnostic accuracy. This meta showed that the diagnostic accuracy of multi-index spirometry (92%) was higher than that of single-index (82%) (*P* < 0.001) and also showed an advantage in sensitivity, 85% in multi-index and 77% in single-index. Therefore, from a clinical implementation perspective, PEF is inexpensive, easy to operate, and the patient can use it easily. Still, its diagnostic accuracy rate renders it unsuitable for diagnosing COPD. It is appropriate to be used to follow-up patients with stable COPD. Previous studies have shown that PEF could be regarded as a prediction tool for the prognosis of the disease^30,31^. Large variability in daily PEF indicated instability in the condition and was susceptible to acute exacerbations.

In our study, the PFTs in tertiary hospitals were all conducted by professional technicians while in primary care centers and communities, these were completed by trained doctors, nurses, or physician assistants. The area under the SROC obtained by the portable spirometer in tertiary hospitals was much higher (0.96) as compared to that in primary care centers or communities (0.89). The results show that PFTs performed by trained general practitioners, nurses, or laboratory assistants in primary care centers and communities can effectively identify persistent airflow limitation, however, compared with professional technicians, there is still room for improvement in diagnostic accuracy measurement. Previous studies have demonstrated that at least 90% of subjects can get acceptable and reproducible results under the operation of experienced professional technicians^32^ but this rate is much lower (58.5–71%) for primary care institutions^33–35^. Taken together, the observations suggest the need to strengthen the supervision of the normative diagnosis and treatment of COPD in resource-limited settings. The professional knowledge, reproducibility, and accuracy of PFTs can be significantly improved for practitioners in medical institutions who have undergone standardized training^33^.

The prevalence of COPD differed across countries and regions. In this study, the diagnostic accuracy of portable spirometers conducted in developing countries was superior to developed countries. We examined the composition of two groups and found that the difference may be that the executive place of the portable spirometer in developed countries was a larger proportion of primary care or community, 73.91% (17 articles) in developed countries, and 50% (4 articles) in developing countries. As mentioned above, there was a difference in the quality of PDTs between trained general practitioners in primary cares and professional technicians in tertiary hospitals. Generally speaking, the accuracy of spirometers conducted by professional technicians in tertiary hospitals could meet an acceptable quality, whereas temporary trained operators in primary cares or communities could not meet for so far. Therefore, regardless of countries or regions, we need to strengthen regular training on spirometer operators to turn this situation around, especially operators in primary care.

Although providing useful insights, there are some limitations to the present study. First, although we used subgroup analyses to explore the sources of heterogeneity, the subgroup variables could not offer complete explanations. This suggests that there may be other confounding variables as sources of heterogeneity. Second, only three included studies were randomly assigned the test order. A portable spirometry test usually precedes the traditional one, which may cause a bias in the test order, that is, the learning effect^36^. Our results show that portable spirometers exhibit high accuracy even in the presence of learning effect. Third, in some studies, both PFTs were performed by the same operator so that blinding was not strictly achieved. Finally, the accuracy of the instrument itself, the choice of the target population, the differences in research design, and the operating procedures may also affect the accuracy of results achieved.

In conclusion, portable spirometers have high accuracy in the diagnosis of COPD. Multi-index Spirometer, such as COPD-6 and PIKO-6, shows superior accuracy over single-indicator. Compared with FEV_1_/FVC, FEV_1_/FEV_6_ can be regarded as a viable surrogate indicator for diagnosing COPD. It is worth noting that although portable spirometers are easier to manoeuvre than laboratory spirometries, they also need to be performed under strict quality control. Standardized training for operators should be strengthened to ensure reliable and reproducible measurements. Portable spirometers are characterized by high accuracy, user-friendly, patient-friendly, inexpensive, and portable, making them suitable for primary care use and providing a feasible pathway for early diagnosis of COPD.

## Data Availability

All data generated or analyzed during this study are included in this published article.
